# What is it like to organize a large-scale educational event for fellow students? A qualitative exploration of student participation in curriculum design

**DOI:** 10.1186/s12909-022-03166-4

**Published:** 2022-02-12

**Authors:** Gert Olthuis, Florieke Eggermont, Bas Schouwenberg, Anke Oerlemans, Esther Tanck

**Affiliations:** 1 Department IQ healthcare, Radboud university medical center, Radboud Institute for Health Sciences, PO Box 9101, HB 6500 Nijmegen, The Netherlands; 2 Orthopaedic Research Lab, Radboud university medical center, Radboud Institute for Health Sciences, PO Box 9101, HB 6500 Nijmegen, The Netherlands; 3 Departments Internal Medicine & Pharmacology – Toxicology, Radboud university medical center, Radboud Institute for Health Sciences, PO Box 9101, 6500 HB Nijmegen, The Netherlands

**Keywords:** Medical education, Student participation, Qualitative research, Experiential learning, Cooperative learning

## Abstract

**Background:**

Although students are increasingly involved in curriculum design, empirical research on practices of actual student participation is sparse. The purpose of this study is to explore the experiences of students who collaborated in the organizing committee of a large-scale educational event, the Radboud Student Conference (RSC), for fellow students.

**Methods:**

We conducted three focus group interviews, in which 17 (bio) medical students of three different organizing teams shared their experiences regarding the organization of the large-scale teaching event. The analysis was conducted using thematic content analysis, in which the codes and codebook were constructed on the basis of the data.

**Results:**

The following four themes were derived from the data. 1) Collaboration, which concentrated on fellow students, teachers who were involved as supervisors, and persons outside the organizing team such as caterers, educational support office members, lecturers, physicians and researchers. 2) Planning and division of labor, with students experiencing a mutual dependence and noticing a gradual improvement of their skills. 3) Freedom implies responsibility, which indicted that students experienced a significant freedom to develop the RSC week, but at the same time felt the responsibility to deliver a successful final week of the academic year. 4) Personal development, where students mentioned the opportunity to practice skills that differed from standard (bio) medical electives.

**Conclusions:**

We conclude that (bio) medical students are capable of bearing the responsibility to organize a large-scale educational event. Organizing the RSC was an educational experience in the form of cooperative and experiential learning which contributed to students’ personal development. Organizing the event gave students both a sense of freedom and the responsibility to succeed. Supervision of faculty members seemed a prerequisite, and tended to be supportive rather than guiding.

**Supplementary Information:**

The online version contains supplementary material available at 10.1186/s12909-022-03166-4.

## Background

Students are increasingly involved in designing medical curricula [1, 2]. This article discusses the learning experiences of students who organized a large-scale teaching week for first, second and third year (bachelor phase) (bio) medical students. In 2015, an extensive revision took place of the bachelor curricula of Medicine (330 students per year) and Biomedical Sciences (100 students per year) at the Radboud university medical center (Radboudumc) in Nijmegen. Students of both programs follow partially the same curriculum. Every academic year of 40 weeks closes with a week filled with compulsory and voluntary activities: the Radboud Student Conference (RSC) (see Table 1 for an impression of the program). The general goals of this week are threefold: 1) to enable bachelor students of different years to complete and present their projects; 2) to organize activities that promote target students’ personal development; and 3) to encourage integration and coherence across years of study and programs in order to gain insight into coming academic years. There are no specific learning materials or formative or summative evaluation of the activities of the RSC since the week focuses on the closing of current projects and internships. However, evaluative questionnaires show that the highly interactive and shared closing of the academic year is appreciated by students.

**Table 1:** Radboud Student Conference program (2017–2018)

Monday – Innovation day	Presentation of innovation projects of 1st year students + assessment of their project
Tuesday – Science day	Poster presentation of science projects of 2nd year students + assessment of their project
Wednesday – Conferences	Biomedical sciences: lectures + presentation of 3rd year internships Medicine: program with keynote lecture and workshops
Thursday – Guided tours	On campus and in the hospital
Friday – Generation day	Program for 1st year students and their family: lectures (both academic staff and students) and workshops

Each year the organizing team of the RSC consists of six to nine dedicated (bio) medical students, supervised by three faculty members. These students have to apply for team membership. After selection, which is done by the faculty members through a short interview, students join the elective course of 4 ECTS (see Table 2 for learning objectives). The course consists of bi-weekly group meetings of the organizing students and teachers, from October until the end of June. Progress is discussed and tasks are shared among the members of the team. Students plan additional meetings together and with external partners and divide different roles, such as chairperson and secretary, among themselves. During the course, students are guided and assessed regarding the described learning objectives (see Table 2). For the final examination, students compose a portfolio which is assessed using a rubric based on the learning objectives. The portfolio is discussed in an individual conversation with one of the supervising faculty members, in which the organizing process as a whole is evaluated as well.

**Table 2:** Learning objectives of organizing the Radboud Student Conference

1. You are able to organize a large-scale educational event in a team.	
2. You are able to fulfill a specific role in the team and take final responsibility for a specific part of the program.	
3. You gain insight into additional matters that are important in the organization of a large event (with about 1000 participants) such as budget / finances, (fire) safety, logistic support and public relations.	
4. You are able to manage peers and students.	
5. You are able to communicate clearly and concisely with all parties involved and establish contacts with external partners who can contribute to the organization of the activities.	
6. You can work systematically.	
7. You are able to promote good cooperation within the team, for example by being open to feedback from others, by giving constructive feedback to your team members, and by behaving professionally towards the team and the tasks.	

The objective of our current study is to explore the experiences of students who collaborated in the organizing committee of the RSC: what is it like to organize a large-scale educational event for fellow students? Although our study departs from an exploratory point of view, our research question is developed against the background of two relevant theoretical perspectives. In the first place we were interested in experiences of students participating in designing and organizing an educational event such as the RSC. Although students are increasingly involved in curriculum design [1, 2], empirical research on practices of actual student participation is sparse [3]. If we look at the participation of the organizing team of students more closely from the perspective of the eight rung ladder of student participation in curriculum design [1, 4], at least two participatory levels are involved (Table 3).

**Table 3:** Ladder of student participation in curriculum design, conform [1]

**8**	**Students in control**	***Co-creation***^***a***^
**7**	**Partnership – a negotiated curriculum**	***Co-creation***
**6**	**Students control of some areas of choice**	***Co-creation***
**5**	**Students control of prescribed areas**	***Co-creation***
**4**	**Wide choice from prescribed areas**	***Participatory design***^***b***^
**3**	**Limited choice from prescribed areas**	***Participatory design***
**2**	**Participation claimed, tutor in control**	***Design based research***^***c***^
**1**	**Dictated curriculum – no interaction**	***Design based research***

^a^Co-creation entails a close collaboration between students and teachers, with a focus on empowering students to actively engage in the educational design

^b^Participatory design is a collaboration between teachers and students in which educational innovations are tailored to learners and context to improve quality and ensure use and usability

^c^Design based research aims to develop answers to educational problems and refine theories. Students are not central actors but may provide input

Particularly in the first years, organizing the RSC was a matter of ‘Partnership – a negotiated curriculum’. This seventh rung of the ladder implies that students had substantial influence in designing the teaching week [4], taking into account a few general requests such as creating the possibility to conclude first and second year projects and a conference for third year biomedical students (see Table 1). The fifth rung of ‘Students control of prescribed areas’ is also relevant. Here, students have some choice and influence and this level indicates that specific areas of the curriculum are designed and controlled by students [4]. Particularly in the later editions of the RSC, the outline of the program was more or less established (Table 1).

In the second place, our exploration was based on the assumption that exploring students’ experiences of organizing such a large-scale educational week could provide insight in the way in which the concept of the ‘zone of proximal development’ (ZPD) takes shape in educational practice. This zone was defined by Vygotski as ‘the distance between the actual development level as determined by independent problem solving and the level of potential development as determined through problem solving under adult guidance of in collaboration with more capable peers’ [5]. The ZPD is to be understood as a metaphorical space, in which the additional potential for learning from the interaction with other agents (e.g., peers, staff, guest lecturers) and structures (e.g., an organizing team, a university medical center, research institutes) is defined [6]. It is the learning that happens when students are in a situation with a task that is slightly too hard for them to do on their own, but simple enough for them to do with assistance [7].

## Methods

To explore the experiences of students who organized the RSC we planned three focus group interviews. Focus group interviews use the interaction among participants to help explore and clarify participants’ views and experiences [8]. Ethical approval was sought from the Research Ethics Committee of the Radboud University Nijmegen Medical Centre (registration number: 2020–7233), who determined ethical approval was not required under Dutch law. All methods were carried out in accordance with the relevant guidelines.

The organizing teams of 2015/16, 2016/17, and 2017/18 were informed by email about the objective of the study, and were invited to participate. The focus group interviews took place in a meeting room of the university medical center. After the interview, the students received a five euro gift voucher for the hospital restaurant as a token of appreciation for their participation.

An interview guide with open-ended questions was developed in collaboration with the staff involved (see Table 4). Two interviews were held in February 2018 and one in December 2018. The focus group interviews were led by a moderator (AO), who was not involved in organizing the RSC and is an experienced qualitative researcher. Each interview commenced by explaining the goal of the study, and a brief introduction of the moderator and the participants. We stressed that participation in this study was voluntary and withdrawal was possible at any time. The interviews were audio-recorded and transcribed verbatim. The anonymity of participants was maintained in the transcripts. All participants signed an informed consent form.

**Table 4:** Key questions of the focus group interviews

• You organized a large-scale teaching week as a team. How do you look back on that?	
• You had a specific role in the team and had a particular responsibility with regard to a part of the program. How did you fulfil that role? What would you do differently next time?	
• How did you experience managing peers?	
• How do you look back on the collaboration with teachers?	
• What did you learn about communication because of your involvement in organizing the RSC?	
• What was the most important lesson you learned during the organization of the RSC?	

The focus group transcripts were coded using ATLAS.ti 8.3 (ATLAS.ti Scientific Software Development GmbH). The analysis was conducted using thematic content analysis, in which the codes and codebook were constructed on the basis of the data [9]. All interview transcripts were carefully read by FE, ET, and GO. One interview transcript (2015/16) was independently coded by FE and ET, after which FE, ET and GO discussed any discrepancies until consensus was reached. The two other transcripts were coded by one researcher (FE), with four consensus meetings together with ET and GO.

This study is an exploration of the experiences of three student teams. No data saturation was intended. To increase the trustworthiness of the findings, independent researchers AO and FE were involved in the collection and analysis of the data and the researchers practiced self-reflexivity throughout the process to reduce possible bias of their involvement (ET, GO) in organizing the RSC [10].

## Results

In three focus group interviews a total of 17 students discussed their experiences regarding the organization of the RSC (see Table 5). We identified the following four themes as central in the interviews, highlighting the experiences of the students: 1) Collaboration; 2) Planning and division of labor; 3) Freedom implies responsibility; and 4) Personal development. See Additional file 1 for an overview of themes, codes and representative quotations.

**Table 5:** Overview of participants

	2015–2016		2016–2017		2017–2018	
	Organizing students, n	In focus group, n	Organizing students, n	In focus group, n	Organizing students, n	In focus group, n
**Total**	6	4	9	7	7	6
**Gender, n**						
Female	4	3	6	4	5	4
Male	2	1	3	3	2	2
**Study, n**						
Biomedical Sciences	0	0	4	3	5	4
Medicine	6	4	5	4	2	2

1. Collaboration

The participating students had to collaborate with many persons to organize the RSC. This collaboration concentrated on fellow students, teachers who were involved as supervisors, and persons outside the organizing team such as caterers, educational support office members, lecturers, physicians and researchers.

Firstly, the students collaborated with other students within the organization committee. Most students did not know each other before joining the RSC committee. They mentioned it was important to become acquainted with each other’s manner of working and to find ways to communicate effectively. This enabled a smoother collaboration and made it easier to ask each other for help or feedback. The participants mentioned they trusted each other with tasks and responsibilities. However, they also indicated that they encountered difficulties in organizing the work, as collaboration was not always smooth. Such difficult collaboration resulted in educational moments, since, at a certain point, students had no other option than to solve the problem together. Participants reported that this taught them how to discuss and deal with such difficulties. In general, however, interviewees mentioned that collaboration within the organization team went well, and that it was fun to work together so intensively towards a specific goal, the RSC. Most participants developed a friendship due to the intensive long-term collaboration with each other.

Secondly, the students collaborated with faculty staff who were involved as supervisors within the organizing committee. Participants indicated they enjoyed collaborating with them and agreed that the teachers were approachable. The students experienced it as working together with peers. However, there were some ambiguities about the intensity of the contact. Some students indicated that they had intensive contact with the teachers, whereas others mentioned there was relatively little contact, as they had expected that teachers would check everything they were doing. Participants reported that they experienced it as helpful that teachers mediated when conflicts emerged within the group, but also if external parties did not keep to the agreements that were made, or if authority was needed.

Thirdly, the students collaborated with teachers and other persons outside the organization committee, for example to organize lectures or workshops, and arrange catering or a party venue. Some students indicated that they were surprised to have contact with so many persons for the organization of the RSC. Participants indicated that they learned how to engage with such external contacts and that clear communication was important. They experienced the importance of making clear agreements with persons involved in the RSC week and how these external contributors to the week can be persuaded to participate. The students reported that communication usually started quite formally, but became more informal after having more contact. Students also encountered difficulties and stress when collaborating with external parties. They encountered individuals who did not respond or did not want to participate. Some of the agreements were not fulfilled which required the students to find ad hoc solutions or ask for help from the supervisors.

2) Planning and division of labor

The participating students reported that planning and division of labor among team members was an important part of the RSC organization. Participants brought forward two more specific experiences in this regard. Firstly, they mentioned that they experienced a complicating aspect of planning: as a team member they depended on input and cooperation from others. Secondly, the participants noticed that their planning skills improved during the organization period. As team members they learned how to create a more realistic planning, how to prepare for meetings, and how to work with an agenda for those meetings. Stimulated by the supervisors, the students collectively made a task division of chairperson, secretary and treasurer at the start of the organization period. They also assigned responsible team members to specific RSC days or activities. Some students indicated that they chose a role that suited them because, for example, they had prior experience as treasurer, whereas others chose a specific role with the aim of improving a certain skill. Participants mentioned they experienced growth in their specific role within the team. According to the students, the division of tasks and responsibilities and the interdependence between students led to a strong team spirit with team members helping each other where possible.

3) Freedom implies responsibility

Participants reported they experienced a significant freedom to develop the RSC week, but at the same time felt the responsibility to deliver a successful final week of the academic year. Participants enjoyed this combination of freedom and responsibility and indicated that they felt a growing sense of responsibility as the organizational process progressed. A recurring subject in the interviews was the stress that came with this sense of responsibility. Through division of tasks and roles, every student became responsible for a part of the organization. Usually tasks were assigned to duos, allowing them to share responsibilities with each other and with the teachers. Particularly the first organizing team (2015/2016) agreed that they experienced the degree of freedom and the lack of boundaries or guidelines to organize the event as stressful. The participants who organized the RSC in later years experienced this as less stressful as they had examples of how the previous editions were organized. The majority of the participants indicated that they had learned a lot because of the freedom they experienced. The students in the later years also indicated that it was a valuable learning experience to have some degree of freedom to adapt and organize the RSC.

4) Personal development

Most participants emphasized the importance of organizing the RSC for their personal development. The participating students mentioned that one of the reasons for choosing this elective was that it differed from standard (bio) medical electives. This particular elective did not concern standard medical knowledge of biomedical skills, but offered an opportunity to practice skills relevant for organizing a large-scale event. This generated new learning experiences. The participating students reported several relevant educational moments, such as the ability to develop new activities for the RSC, plan activities while the number of participants was unknown, learning about the organizational context of a university hospital and medical education, developing websites, and how to handle last minute changes. Additionally, participants indicated that they had acquired more general skills regarding collaboration, communication, taking responsibility, planning, and coping with stress. Furthermore, it was a learning experience for the participants to be seen as colleagues rather than regular students.

## Discussion

Our study explored the experiences of (bio) medical students who organized a large-scale educational event for fellow students. We investigated the experiences of students who organized editions of the RSC in 2015/16, 2016/17, and 2017/18 in three separate focus group interviews. From the data we derived four central themes: 1) Collaboration; 2) Planning and division of labor; 3) Freedom implies responsibility; and 4) Personal development. In this section we will further interpret our results and discuss them in two steps. First we interpret our results in the context of other research on student participation and engagement. Second, we elaborate on the ZPD which formed the theoretical background of our study and relate our results to experiential and cooperative learning.

### Participation

The key benefits of active student participation that were considered in a recent commentary are enhanced student satisfaction, meta-cognitive understanding of learning and teaching processes, enhanced student-staff relationships, and development of personal, professional, and academic competences of students [2]. Our results showed that particularly the development of various competences was part of the experiences of the organizing students, in addition to enhanced student-staff relationships and student satisfaction. An improved meta-cognitive understanding of learning and teaching processes is not in question since the experiences of participants did not concern the development of actual (bio) medical education but concentrated on organizing an educational week. Successful student engagement also requires an institutional culture that empowers the students’ voice and their activities, a clear framework that describes the expected relationship between students and faculty, and effective communication routes between students with peers and faculty [3]. In the RSC this basis was provided by the structure of bi-weekly meetings and division of roles and tasks at the start of the organization period.

Student participation comes in various forms: as a student work group of medical education [11], as a partnered education governance model with a student committee providing input for undergraduate medical education [12], as students functioning as module co-directors in curriculum change [13], or a Student Curricular Board that is fully integrated in ongoing curriculum management and quality improvement processes [14]. There is however little research on actual experiences of students who participated in curriculum design and the development of medical education [3]. A survey among students who engaged in a work group to contribute to the design and improvement of an undergraduate curriculum led to some results that are similar to our study [11], particularly the enhancement of skills with regard to decision-making, communication with fellow students and problem tackling. Another example concerns the engagement of German students as module co-directors in curriculum development [13]. This initiative of co-development was investigated in a mixed-methods study of surveys and a focus group interview with eight students who participated as co-directors. Although engagement in the development of a curriculum differs from organizing a large-scale educational week, there are some striking similarities between the experiences of the co-directing students and our participants. Just like the German students, our participants experienced a challenge in dealing with hierarchy in the university medical center and negotiation processes both within the team and outside the team with external contacts. And just like the German students our participants experienced the value of being well-prepared for meetings, having the possibility and responsibility to generate own creative new ideas and solutions, and mutual support between team members.

### Experiential and cooperative learning

We started our exploration of student experiences from the assumption that organizing the RSC took place in a ZPD: it is too hard for students to do on their own, but simple enough to do with assistance [7]. We believe the analysis of the results acknowledges this theoretical starting point and provides an outlook on two aspects of learning that take place within the ZPD. First, the experiences of our participants showed that the learning that took place during the organization of the RSC is a form of experiential learning. This type of learning starts from three assumptions: 1) learning is situated (it took place within the social context of the organizing team); 2) learning can be viewed as either individual and collective (interaction between students and staff is fundamental in experiential learning); and 3) the learning is triggered by authentic practice-based experiences (organizing this closing week was real, there was something at stake) [6].

Second, we argue that our data show that organizing a large-scale event such as the RSC can be understood as a form of cooperative learning. Cooperative learning involves students working together to achieve common goals or achieve group tasks that they would be unable to complete by themselves [15]. The most distinct characteristic of cooperative learning seems to be the interdependence between group members, who can reach their goals if all members cooperate [15–17]. The five elements [15, 17] that distinguish cooperative learning from other forms of group work can easily be applied to the experiences of students who organized the RSC. In addition to the element of positive interdependence which emerges from establishing group goals such as organizing a large-scale teaching week and the division of tasks and team roles, the group process in which decisions are made and reflection on their collaboration and progress takes place is a second element of cooperative learning. A third element is the importance of face-to-face interaction to stimulate mutual discussion and trust within the team. This relates to a fourth feature of cooperative learning which concerns the opportunity to practice and improve interpersonal skills, such as active listening, sharing ideas, providing constructive comments on others’ ideas, making shared decisions, and managing disagreements. A fifth key element in cooperative learning is individual accountability; students have their own responsibility with regard to completing their personal tasks while also contributing to the collective group effort ensuring that others complete their tasks. Apart from these five elements that can easily be recognized in our results, teaching staff plays a facilitating role in cooperative learning. They have a role in structuring the group of students thus stimulating the process of cooperative learning, promoting interaction, dialogue and collaboration within the group and – if necessary – mediate in resolving problems [15].

Our study provided a qualitative exploration of student participation in curriculum design. A strength is that our exploration provides an in-depth look at the actual experiences of students who organized a large-scale educational event. Also, an independent researcher (AO) moderated the focus group interviews to warrant a safe and open conversation between the participants. A third strength is that we have included participants from three consecutive organizing teams, thus ensuring a more solid validity of our data. Three factors may limit the generalizability of our study. First, the focus group interviews were conducted in 2018. Recall bias may have occurred, since for the students who organized the RSC in 2015–2016 participation was some years ago. Second, not all students of the various organization committees participated in the focus group interviews (see Table 2). Selection bias may have occurred. Third, group dynamic within a focus group interview may have inhibited critical evaluation of each other.

## Conclusions

In conclusion, (bio) medical students are capable of bearing the responsibility to organize a large-scale educational event. Our study showed that student participation in educational development creates an effective learning environment. Organizing the RSC was an educational experience in the form of cooperative and experiential learning which contributed to students’ personal development. Students also experienced improved planning skills as well as positive and negative aspects of collaboration with peers and faculty members. Organizing the RSC gave students both a sense of freedom and the responsibility to succeed. Supervision of faculty members seems a prerequisite, and tends to be supportive rather than guiding. We recommend supervising teachers to have confidence in their students, to monitor and structure the group process and promote positive interaction between the students involved.

## Supplementary Information

**Additional file 1.** Representative quotations of study participants.

## Data Availability

The datasets generated and/or analysed during this study are not publicly available due to privacy and confidentiality reasons but are available from the corresponding author on reasonable request.

## Abbreviations

RSC: Radboud Student Conference
ZPD: Zone of Proximal Development
