# Supplementary material for: What is it like to organize a large-scale educational event for fellow students? A qualitative exploration of student participation in curriculum design
Source: BMC Med Educ. 2022 Feb 12;22:95. doi: 10.1186/s12909-022-03166-4 (PMC8841077; doi:10.1186/s12909-022-03166-4)
Supplement: Supplementary file 1 — Additional file 1. Representative quotations of study participants. [file 12909_2022_3166_MOESM1_ESM.docx]

**Appendix 1** *–* Representative quotations of study participants

| **Theme** | **Codes** | **Representative quotations** | **Year** |
| --- | --- | --- | --- |
| **Collaboration** | | | |
| Collaboration with students | Important to become acquainted with each other | You don’t really know what to expect. But when I look back now I think: wow look at what we have accomplished so far. We didn’t know each other, nine persons is quite a lot, also to hold meetings with. | 2015-2016 |
|  | Trust | You cannot organize the whole week by yourself so if [student] decides that we will do things this way, you do it that way because you trust her to make correct time schedule. | 2016-2017 |
|  | Educational moments | When collaborations aren’t easy or things just aren’t happening, but they ought to happen. That as a team you make sure that they happen, so to speak. […] Or that it will at least be discussed. […] Yes, at a certain moment you cannot avoid discussing it. […] And then you talk about it with a teacher. Like, what should we do with this? | 2017-2018 |
|  | Friendship | Because we always started with just chatter and at some point we became more serious. And as soon as our meeting ended, we often stayed and hung out. And that resulted in something like friendship. | 2017-2018 |
| Collaboration with teachers | Approachable teachers | But I did enjoy it, because, yes they [the teachers] were just very approachable, and you could just communicate normally with them. | 2015-2016 |
|  | Teachers help fixing conflicts | Because on the day itself, I remember, we ran into something of a problem, and [teacher] just solved it. | 2015-2016 |
| Collaboration outside the organization committee | The importance of clear communication | And that you must clearly state what you want. And stop beating around the bush. | 2017-2018 |
|  | Formal versus informal communication | Of course you start off very formally and it’s up to the other person to determine whether you continue that way or not. […] I noticed that, when things get more informal, it is easier. | 2017-2018 |
| **Planning and division of labor** | | | |
| Planning | Dependent on input and cooperation from others | Especially in the beginning we made a very precise planning like from then until then we do this and from then until then we do that. […] But yes, even though you make a planning, for such a large project you depend on so many people, and very often someone doesn’t respond or someone is not available or something is cancelled and then everything in the planning shifts, so you never stick to the first planning. | 2016-2017 |
|  | Educational moments | Yes, well, I already was a good planner, but this is different planning. Now you need to plan around people and with people. And that is quite difficult. Because everybody is always busy. […] And that is something you have to learn to deal with. And that is also … now I am … now I can deal with that. In the beginning it is very difficult because you‘re fresh out of high school … yes. Nobody is really busy there, everybody can always contribute. And now suddenly, you really need to take others into account. | 2017-2018 |
| Division of labor | Role that fits you versus role to improve certain skill | Interviewer: And do you mainly choose things of which you know “I am good at that” or more like “I want to learn that”?  Both is possible. You need to set your learning objectives, so if you always do what you are good at, you won’t make progress. | 2016-2017 |
| **Freedom implies responsibility** | | | |
|  | Freedom | I did really enjoy that we had a lot of say. We were allowed to keep it close to the student. […] So I was very motivated. You had to be available a lot, also during the RSC itself, and the responsibility was actually great. | 2015-2016 |
|  | Stress that comes with responsibility | I did experience that pressure at a certain moment, because you really need to make sure that the organisation is okay and yes, you are responsible for the part that you take care of, and yes, sometimes things did not go as you wanted. | 2015-2016 |
|  | Sharing responsibility | You’re working together, like on Wednesday you’re working with [student], then you can really talk things over and then yes, you share the responsibility a little. I enjoyed that. I think, we divided the chairs for the Monday, Tuesday, Wednesday, Thursday and Friday but in the end it is still done together with everyone. | 2016-2017 |
| **Personal development** | | | |
|  | Different from standard education or committees | But it was something completely different from other courses, […] and usually it is medicine-related, whether it is about how the human body works or something like that, or about health … It is all comparable but this was something completely different I thought. Because you are really developing education. | 2015-2016 |
|  | Educational moments | As we mentioned before, the planning, the organization, I think I learned a lot from that. Also how a hospital is organised, who directs what and how education takes shape, who is responsible for the costs, or responsible for giving days off in those two weeks, and those are interesting things to know. | 2016-2017 |
